# Chinese herbal compound combined with western medicine therapy in the treatment of plasma cell mastitis: A protocol for systematic review and meta-analysis

**DOI:** 10.1097/MD.0000000000022858

**Published:** 2020-10-30

**Authors:** Jindan Zhang, Jianzhong Xu, Jiao Zhang, Yun Ren

**Affiliations:** a Changzhi City People's Hospital, Changzhi, Shanxi province, China.

**Keywords:** chinese and western medicine, meta-analysis, plasma cell mastitis, protocol, randomized controlled trials

## Abstract

**Background:**

Plasma cell mastitis (PCM) is a benign suppurative disease of the breast based on the expansion of mammary ducts and infiltration of plasma cells. It is relatively rare clinically, and its main manifestations include nonperiodic breast pain, nipple discharge, areola lump, nipple depression, nipple fistula, among others. Modern medicine is mainly surgical treatment, which is easy to recur. The clinical practice shows that the overall treatment of patients with TCM syndrome differentiation using oral medicine combined with western medicine therapy, combined internal and external treatment, can significantly improve the curative effect, prevent recurrence, has a certain therapeutic advantage, but lack of evidence of evidence-based medicine. The purpose of this study is to study the efficacy and safety of oral traditional Chinese medicine (TCM) combined with western medicine therapy in the treatment of PCM.

**Methods:**

Use computer to retrieve English databases (PubMed, Embase, Web of Science, the Cochrane Library) and Chinese databases (CNKI, Wan Fang, VIP, Chinese biomedical database), from the establishment of database to September 2020, for randomized controlled trials(RCTs) of oral TCM combined with western medicine therapy in the treatment of PCM, two researchers independently extracted the data and evaluated the quality of the included research, and meta-analysis was conducted on the included literatures using RevMan5.3 software.

**Results:**

This study evaluated the efficacy and safety of oral TCM combined with western medicine therapy in the treatment of PCM from the aspects of effective rate, symptom score, recurrence rate, adverse reaction rate, and patient satisfaction.

**Conclusion:**

This study will provide reliable evidence-based evidence for the clinical application of oral TCM combined with western medicine therapy in the treatment of PCM.

**Ethics and dissemination:**

The purpose of this study is to sort out and analyze the literature. This systematic review also does not involve endangering participant rights. Ethical approval was not required. The results may be published in a peer-reviewed journal or disseminated at relevant conferences.

**OSF Registration number:**

doi 10.17605/OSF.IO/K9A78.

## 1 Introduction

Plasma cell mastitis (PCM), also called mammary duct ectasia or occlusive mastitis, based on the pathological basis of mammary duct dilation, massive infiltration of plasma cells and lymphocytes, and the clinical manifestations are nonperiodic breast pain, nipple discharge, areola area lump, nipple depression, breast abscess during non-feeding, nipple fistula, and so on, mainly occurs in females at nonpregnant and nonlactating stages,^[1,2]^ occasionally seen in men. It is relatively rare in clinical practice, and its incidence rate accounts for 1.41%∼5.36% of the breast diseases in the same period, showing an increasing trend in recent years.^[3,4]^ Since its clinical manifestations lack specificity, it is easy to be misdiagnosed and mistreated, often confused with mastitis, breast cancer, and other diseases.^[5,6]^ Because its infection repeatedly attacks, easy to form sinus or fistula, there is no effective drug treatment, while surgical treatment used more often, but still repeated attacks after the operation, protracts course of disease, and even leads to total breast resection, causing physical and mental trauma of patients, making the disease a difficult problem in breast surgery,^[7,8]^ so it is necessary to seek new treatment.

PCM belongs to the category of “Fen ci xing ru yong” (comedogenic mammary abscess) in Chinese medicine. In 1985, the name was first put forward by Gu Baihua and published in *Practical Chinese Medicine Surgery*,^[9]^ pointing out that the pathogenesis was related to congenital inverted nipples, discordation of liver and spleen, obstruction of breast collaterals, and interaction of phlegm and blood stasis. Clinically, according to qi stagnation, phlegm coagulation, blood stasis, heat poison and other pathological factors, taking targeted oral Chinese medicine for syndrome differentiation treatment, or combined with various traditional Chinese surgical therapy, can reduce local symptoms of breast, reduce the change of breast appearance, reduce the recurrence rate after western medicine therapy, has good clinical efficacy.

At present, the results of several randomized controlled studies have shown that the treatment of PCM with oral traditional Chinese medicine (TCM) combined with western medicine therapy can avoid unnecessary surgery to damage the breast, enhance the therapeutic effect, and significantly reduce the recurrence rate. However, there are differences in the research scheme and curative effect of each clinical trial, which leads to the uneven results and to some extent affects the promotion of the therapy. Therefore, this study plans to systematically evaluate the efficacy and safety of oral TCM combined with western medicine therapy in the treatment of PCM, and provides a reliable reference for the clinical application of TCM compound in the treatment of PCM.

## 2 Methods

### 2.1 Protocol register

This protocol of systematic review and meta-analysis has been drafted under the guidance of the preferred reporting items for systematic reviews and meta-analyses protocols (PRISMA-P) and it has been registered on open science framework (OSF) on September 21, 2020.(Registration number: DOI 10.17605/ OSF.IO / K9A78).

### 2.2 Ethics

Because this study does not require patient recruitment, as well as patients’ personal information, it does not require the approval of the ethics committee.

### 2.3 Eligibility criteria

#### 2.3.1 Types of studies

We collected all available randomized controlled trails (RCTs) on oral TCM combined with western medicine therapy in the treatment of PCM, regardless of blinding, publication status, and region, but language was restricted to Chinese and English.

#### 2.3.2 Research subjects

Among patients with definite diagnosis of PCM, nationality, race, age, sex, course of disease, and so on were unlimited.

#### 2.3.3 Intervention measures

The treatment group was treated with oral TCM combined with western medicine therapy. The control group was treated by western medicine therapy only. There shall be no restrictions on the types, doses, frequency, and course of treatment of Chinese compound medicines taken internally and there was no limit to the methods of western medicine.

#### 2.3.4 Outcome indicators

1. Primary outcome: the overall effective rate; total effective rate =(cure number + effective number)/ total number × 100% (*Diagnostic and curative effect criteria of TCM diseases and syndromes* of National Administration of TCM 2000: Evaluation Criteria for the efficacy of PCM,^[10]^ Cure: no recurrence within 6 months after withdrawal of the drug, breast lump has been significantly reduced and pain has been significantly dissipated. Effective: breast lumps and pain have been locally dissipated more than half; Invalid: no improvement or further exacerbation of clinical symptoms.)
2. Secondary outcomes: ① Symptom score; ① Recurrence rate; ① Incidence of adverse reactions; ① Patient satisfaction.

### 2.4 Exclusion criteria

1. Studies published repeatedly
2. Studies which were abstracts or study whose data was incomplete, and relevant literature cannot be obtained after contacting the author
3. Studies with obvious data errors
4. Interventions combined with other TCM therapy, such as external application of TCM, fire needle drainage, surgical hanging, acupuncture, and moxibustion
5. The intervention measures were for external use of TCM
6. The patient was a pregnant or lactating woman
7. Studies without relevant outcome indicators

### 2.5 Search strategy

With “zhong xi yi jie he”(Chinese and western medicine), “zhong yao fu fang”(Chinese herbal compound), “jiang xi bao xing ru xian yan”(plasma cell mastitis), “ru xian dao guan kuo zhang zheng”(Mammary duct ectasia), etc, as Chinese retrieval words, retrieve in Chinese database, including CNKI, Wanfang Data Knowledge Service Platform, Chinese Journal Service Platform (VIP), Chinese Biomedical Database; Use “traditional Chinese medicine,” “Chinese medicine,” “Integrative Medicine,” “plasma cell mastitis,” “mammary duct ectasia,” and so on as English search terms, retrieve in English databases, including PubMed, Embase, Web of Science, the Cochrane Library. The retrieval time was from the establishment of the database to September 2020, and all domestic and foreign literatures on the treatment of PCM by oral TCM combined with western medicine therapy were collected. Take PubMed as an example, and the retrieval strategy is shown in Table 1.

**Table 1:**
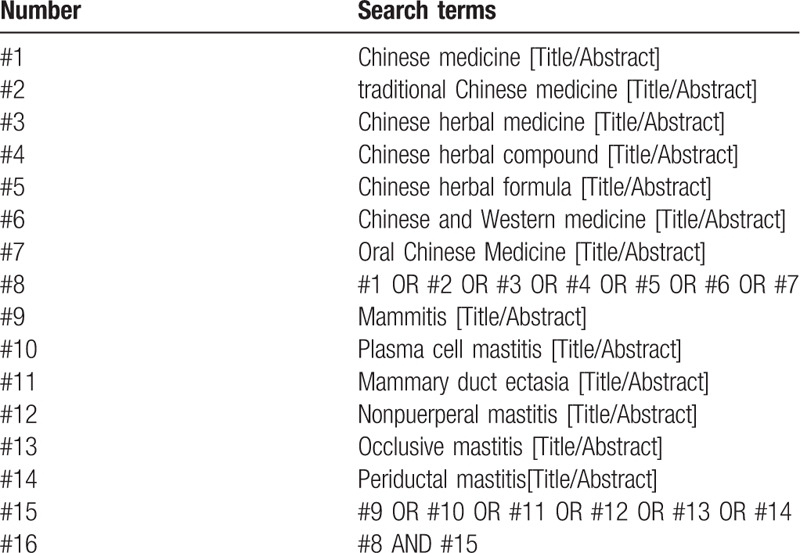
Search strategy in PubMed database.

### 2.6 Data screening and extraction

**Figure 1:**
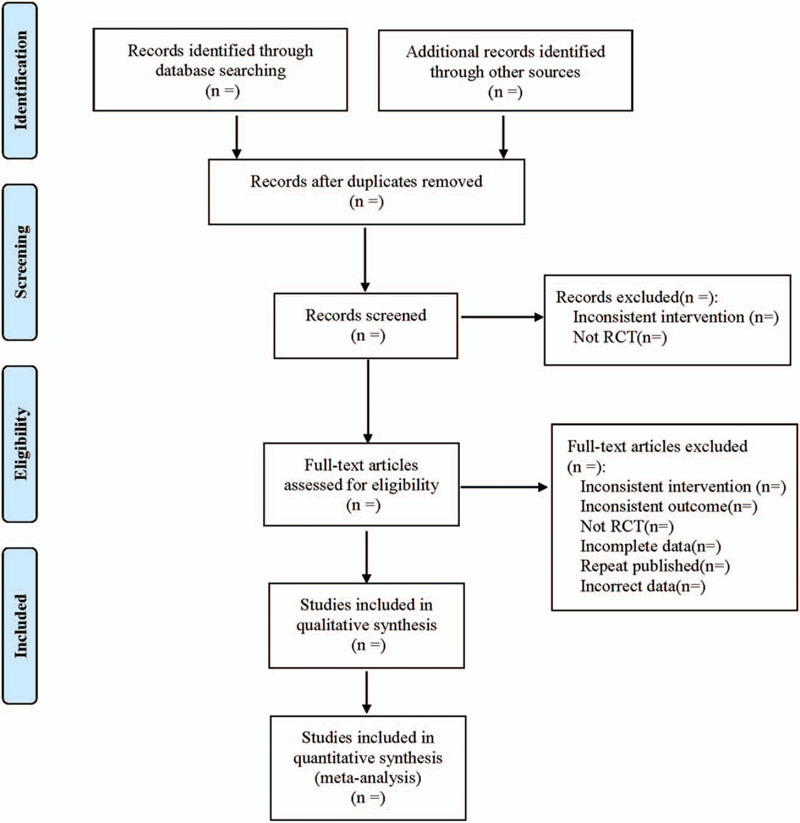
Flow diagram.

We referred to the method of research selection in Cochrane Collaboration System Reviewer Manual Version 5.0, according to the PRISMA flow chart; 2 researchers used the EndNote X9 document management software to independently screen the literatures according to the above inclusion and exclusion criteria and check each other, that if there were different opinions, a third party was needed to be negotiated to resolve the differences. At the same time, use Excel 2013 to extract relevant information, including: ① Clinical study (title, first author, year of publication, sample size, sex ratio, mean age, mean course, stage); ② Intervention measures (type, core drugs, dosage, frequency, course of treatment in treatment group; the specific plan of western medicine treatment in treatment group and control group); ③ Risk bias assessment elements in randomized controlled trials; ④ Outcome indicators. The literature screening process is shown in Figure 1.

### 2.7 Literature quality assessment

The Cochrane collaboration's tool for assessing risk of bias will be used to do the risk of bias assessment of included studies. According to the performance of the studies included in the above evaluation items, the 2 researchers will give 3 judgments (low-risk, unclear, high-risk judgments) one by one, and then carry out cross-checking after completion respectively. If there may be some disagreement, they will have a discussion. If the parties cannot reach an agreement, discussions will be held with a third party researcher.

### 2.8 Statistical analysis

#### 2.8.1 Data analysis and processing

The RevMan 5.3 software provided by the Cochrane Collaboration will be used for statistical analysis. ① For the dichotomous variables, relative risk is selected as the statistic; for the continuous variables, weighted mean difference is selected when the tools and units of measurement indicators are the same, and standardized mean difference is selected when with different tools or units of measurement, and all the above are represented by effect value and 95% confidence interval.② Heterogeneity test: The heterogeneity was determined by *χ*^*2*^ and *I*^*2*^ values. *I*^*2*^ value is used to quantitatively evaluate the inter-study heterogeneity. If *I*^*2*^ ≤50%, the heterogeneity is considered to be good, and the fixed-effect model is adopted. If *I*^*2*^ > 50%, it is considered to be significant, the source of heterogeneity will be explored through subgroup analysis or sensitivity analysis. If there is no significant clinical or methodological heterogeneity, statistical heterogeneity is considered to exist, and random-effect model is used for analysis. Descriptive analysis will be used if significant clinical heterogeneity exists between the 2 groups and subgroup analysis cannot be available.

#### 2.8.2 Dealing with missing data

If data are missing in the article, contact the author by email for more information. If the author cannot be contacted, or the author has lost the relevant data, descriptive analysis will be carried out, not meta-analysis.

#### 2.8.3 Subgroup analysis

Subgroup analysis was carried out according to the treatment group of western medicine assisted Chinese herbal compound and western medicine treatment only. Subgroup analysis was carried out according to the clinicopathological type of PCM, which can be divided into 4 subgroups: spill type, lump type, abscess type, fistula type. Subgroup analysis was carried out according to the course of treatment. Subgroup analysis was carried out according to different surgical schemes.

#### 2.8.4 Sensitivity analysis

To test the stability of meta-analysis results of indicators, sensitivity analysis will be carried out in a one-by-one elimination method.

#### 2.8.5 Assessment of reporting biases

If no <10 studies were included in an outcome measure, funnel plots were used to assess publication bias. Moreover, Egger and Begg test were used to assess the evaluation of potential publication bias.

#### 2.8.6 Evidence quality evaluation

Using the Grading of Recommendations Assessment, Development, and Evaluation (GRADE) to assess the quality of evidence. It contains 5 domains (bias risk, consistency, directness, precision, and publication bias). And the quality of evidence will be rated as high, moderate, low, and very low.

## 3 Discussion

Western medicine believes that mammary duct dilatation and plasma cell infiltration are the key pathological processes of PCM. Studies have shown that ICAM-1 in mammary duct epithelium and endothelial cells play an important role in PCM inflammation.^[11]^ However, at present, scholars have not reached a consensus on the etiology of PCM, most of the debates focus on immune,^[12]^ nipple congenital malformation, and so on, such as Liu et al show that IL-6/STAT3 signaling pathway is activated in PCM, and it is speculated that it plays an important role in the pathogenesis of PCM. For treatment, western medicine advocates early and complete resection of lesions and correction of nipple retraction is^[13]^ effective treatment. And it has been reported that the cure rate after surgical resection can reach 79%,^[14]^ but often repeated attacks after surgery, protracts course of disease, cause huge trauma in the shape of the breast, and even leads to total mastectomy, causing physical and mental trauma to patients, especially female patients. Therefore, the disease has become a difficult diagnosis and treatment of breast surgery. Traditional medicine holds that the pathogenesis of PCM is: at the basement of congenital nipple depression deformity and the long-term stagnation of liver-qi and blood stasis, internal heat generates, heat burns the flesh, pus forms, and fistula forms after ulceration, or due to liver-qi stagnation and spleen deficiency, the phlegm forms and accumulates with blood stasis, internal heat generates and burns meat into pus, then heat poison stays after ulceration, causing the disease cannot be healed for a long time.^[15]^ Chinese medicine treat the disease by stages combined with syndrome differentiation treatment. The acute stage and subacute stage are classified as liver meridian heat accumulation type, using Chaihu Qinggan Decoction, Kaiyu Powder, Tounong Powder, Gualou Niubang Decoction, and so on; chronic stage is the remaining poison type, choose Yanghe Decoction, Tuoli Xiaodu Powder, and so on. High-frequency TCMs for PCM treatment include Pugongying (*Herba Taraxaci*), Chaihu (*Radix Bupleuri*), Danggui (*Radix Angelicae Sinensis*), Shenggancao (*Radix Glycyrrhizae*), Zaojiaoci (*Spina Gleditsiae*), Chishao (*Radix Paeoniae Rubra*), Danshen (*Radix Salviae Miltiorrhiae*), and so on. In TCM theory, Pugongying (*Herba Taraxaci*) is good for breast carbuncle and swollen poison; Chaihu (*Radix Bupleuri*) has the effect of soothing the liver qi and relieving depression and lifting the Yang qi; Danggui (*Radix Angelicae Sinensis*) can tonify blood and activate blood circulation; Shenggancao(*Radix Glycyrrhizae*) can detumescence by detoxification, while warm yang to remove obstruction when baked, so it is suitable for the period of heat toxin and yang deficiency; Chishao (*Radix Paeoniae Rubra*) and Danshen (*Radix Salviae Miltiorrhiae*) can both activate blood circulation and disperse blood stasis, Danshen (*Radix Salviae Miltiorrhiae*) can also cool blood and eliminate carbuncle. Modern pharmacological studies have found that saikosaponin SS has a significant anti-inflammatory effect, which is manifested in inhibiting the release of inflammatory mediators and the growth of granuloma, anti-exudation, and inhibiting the increase of capillary permeability caused by histamine and 5-hydroxytryptamine. At the same time, it also has a significant inhibitory effect on leukocyte migration, connective tissue proliferation and other inflammatory reactions.^[16]^

It is proved that oral Chinese medicine combined with western medicine therapy is effective in the treatment of PCM. Nevertheless, the evidence from RCTs is not consistent. With the increase of clinical trials, it is urgent to systematically evaluate the PCM of oral TCM combined with western medicine therapy. In this study, we will summarize the latest evidence of the efficacy of oral TCM combined with western medicine therapy in the treatment of PCM. This work also provides useful evidence for determining the efficacy and safety of oral Chinese herbal compound combined with western medicine therapy in PCM patients, which is beneficial for both clinical practice and health-related decision makers. However, this systematic review has some limitations. The types, dosage forms, dosage, and composition of Chinese medicine compound used in the study were different, the way of western medicine therapy and the degree of patient's condition were different, and there may be some clinical heterogeneity. The course of disease is also different, and may have a certain impact on the results. Due to the limitation of language ability, we only search English and Chinese literature, and may ignore the research or report of other languages.

## Author contributions

**Data collection:** Jindan Zhang and Jianzhong Xu.

**Funding support:** Yun Ren.

**Literature retrieval:** Jindan Zhang and Jianzhong Xu.

**Software operating:** Jiao Zhang.

**Supervision:** Yun Ren.

**Writing – original draft:** Jindan Zhang and Jianzhong Xu.

**Writing – review & editing:** Jindan Zhang and Yun Ren.
